# Lyotropic Liquid Crystals: A Biocompatible and Safe Material for Local Cardiac Application

**DOI:** 10.3390/pharmaceutics14020452

**Published:** 2022-02-20

**Authors:** Antonia Mancuso, Eleonora Cianflone, Maria Chiara Cristiano, Nadia Salerno, Martine Tarsitano, Fabiola Marino, Claudia Molinaro, Massimo Fresta, Daniele Torella, Donatella Paolino

**Affiliations:** 1 Department of Experimental and Clinical Medicine, University “Magna Graecia” of Catanzaro, Viale Europa snc, 88100 Catanzaro, Italy; antonia.mancuso@unicz.it (A.M.); mchiara.cristiano@unicz.it (M.C.C.); marino@unicz.it (F.M.); 2 Department of Medical and Surgical Sciences, University “Magna Graecia” of Catanzaro, Viale Europa snc, 88100 Catanzaro, Italy; cianflone@unicz.it (E.C.); nadia.salerno@unicz.it (N.S.); c.molinaro@unicz.it (C.M.); 3 Department of Health Science, University “Magna Graecia” of Catanzaro, Viale Europa snc, 88100 Catanzaro, Italy; martine.tarsitano@studenti.unicz.it (M.T.); fresta@unicz.it (M.F.)

**Keywords:** lyotropic liquid crystals, cardiac tissue regeneration, rheological characterization, epicardial application, in vivo studies, biocompatible material

## Abstract

The regeneration of cardiac tissue is a multidisciplinary research field aiming to improve the health condition of the post-heart attack patient. Indeed, myocardial tissue has a poor ability to self-regenerate after severe damage. The scientific efforts focused on the research of a biomaterial able to adapt to heart tissue, thus guaranteeing the in situ release of active substances or growth promoters. Many types of hydrogels were proposed for this purpose, showing several limitations. The aim of this study was to suggest a new usage for glyceryl monooleate-based lyotropic liquid crystals (LLCs) as a biocompatible and inert material for a myocardial application. The main advantages of LLCs are mainly related to their easy in situ injection as lamellar phase and their instant in situ transition in the cubic phase. In vivo studies proved the biocompatibility and the inertia of LLCs after their application on the myocardial tissue of mice. In detail, the cardiac activity was monitored through 28 days, and no significant alterations were recorded in the heart anatomy and functionality. Moreover, gross anatomy showed the ability of LLCs to be bio-degraded in a suitable time frame. Overall, these results permitted us to suppose a potential use of LLCs as materials for cardiac drug delivery.

## 1. Introduction

Cardiovascular diseases are the main cause of death in the world; the majority of fatal events are myocardial infarctions (MI) and the resulting heart failure [1]. Acute myocardial infarction is due to a myocardial necrotic event caused by an unstable ischemic syndrome [2], with blockage or occlusion of the coronary artery [3]. As a consequence of this dramatic event, a portion of myocardial tissue undergoes a process of adverse changes that consist of left ventricular (LV) remodeling. During this pathological process of remodeling, the wall of the heart thins, and a massive loss of cardiomyocytes occurs [4]; these pathophysiological alterations can induce heart failure and in several cases death [5]. To date, the clinical approaches involve mainly traditional pharmacological treatments (as the use of β-adrenergic receptor blocking agents, angiotensin-converting enzyme (ACE) inhibitors and mineralocorticoid-receptor antagonists) [6] and left ventricular assistant devices, but with poor success in terms of individuals’ life expectancy. In this scenario, the concepts of tissue engineering and regenerative medicine are growing increasing interest in the attempt to overcome the limitation of the current pharmacological treatments of heart failure and heart transplantation, which, despite being the only effective approach, remains available to too few patients considering the low organ availability, blood-type compatibility and organ rejection [7]. Regenerative medicine is indeed a field set to make possible the repair and regeneration of organs previously viewed as incapable of regeneration or negligible repair such as the adult heart. To generate new functional myocardium in order to repair the injured one, several approaches have been tested in the past years, with stem cell therapy becoming the most attractive one. Induced pluripotent stem cells [8], in situ reprogramming of non-contractile cells into new cardiomyocytes, as well as embryonic stem cells [9], bone marrow stem cells [10], mesenchymal stem cells and the transplantation of autologous and allogeneic cardiac stem cells (CSCs), have been employed in different animal models of MI and several have been tested in small phase I/II clinical trials in patients with overall modest effects [11]. The explosion of interest in regenerating diseased hearts is being further boosted by advances in nanotechnology, genetics, imaging and other research fields [12,13]. However, the main challenge remains to understand how to harness and enhance the heart’s intrinsic regenerative potential.

To this end, four modern strategies are suggested for promoting cardiac tissue regeneration; first, the direct injection of stem cells/progenitors into the cardiac environment for inducing a direct myocardial delivery; the injection of acellular materials for inducing endogenous repair mechanism; artificial valve replacement; and the implantation of cardiac scaffolds [14].

Among the main exponents of cardiac scaffolds, preformed patches and hydrogels are suggested as valid platforms for the site-specific delivery of drugs and stem cells and for the mechanical support necessary during cardiac regeneration. For example, some research groups have demonstrated that the intramyocardial hydrogel injection is useful to reduce and control the LV remodeling, reducing the necrotic process of damaged myocardium and the expansion of non-contractile regions [15,16,17]. Regardless of the type, the necessary requirements of a hydrogel for cardiac tissue regeneration are stiffness, safety, degradability, compatibility with the host tissue, and if possible, a minimally invasive surgical approach for the implantation [15].

No less important is the gelation time, namely the time necessary to complete the sol-gel transition. An ideal hydrogel scaffold should be applied in liquid form to allow it to adapt to the damaged site and to induce its perfect stratification through an in situ sol-gel transition following specific stimuli (temperature, pH, photoinduction, moisture), in a short time. In detail, the gelation time must be controlled to avoid the gelation that occurs outside the body or during the administration, and at the same time, it must be fast enough to avoid the injected sample that moves from the application site. Ideally, the gelation should be immediate, and occur soon after the contact with myocardial tissue.

Hydrogels responding to UV radiation, temperature or pH have already been proposed for cardiac tissue engineering for stimuli, but there are critical points of concern. For example, light-inducible photo-crosslinking can induce the in situ gelling of an administered polymer solution. The resulting gel is characterized by good elasticity, ability to provide mechanical support and very short gelation time, which are all features required for an effective cardiac hydrogel. Unfortunately, the irradiation necessary to induce the sol-gel transition could negatively impact the human body, causing probable endogenous oxidative damage to DNA and reducing tissue healing [18]. On the contrary, thermosensitive polymers permit obtaining in situ forming gel as a response to the temperature, without using chemical crosslinkers or UV radiation [19]. Normally, the gelation of a thermosensitive polymer solution occurs quickly, but it depends on its composition. For example, Ke et al. [20] proposed a thermosensitive hydrogel for cardiac tissue engineering composed of chitosan and β-glycerophosphate. The gelation time recorded by the authors for their basic formulation was 347 ± 6 s when it was at 37 °C, a too long of a time to obtain perfect gelation in situ without the loss of material due to the flow of biological fluids. For this reason, the authors improved their formulation by adding dextran, thus recording a new in situ gelling time of 10 ± 1 s.

Other authors proposed a pH-sensitive hydrogel, namely hydrogel that becomes gel at a specific pH value [21]. To obtain an efficient in situ gelation of these pH-sensitive gels, it is important to perfectly balance the composition depending on its response to the pH of the environment in which it is located, which is not always constant and precise. In fact, the pH of the cardiac environment in post-MI should be around a pH value of 6 [21,22,23]. So, to guarantee an in situ gelation, the pH-sensitive hydrogel must have a basal pH over 7–8, avoiding showing gelation before the achievement of the application into the site [21].

Starting from the scientific evidence presented and awareness of the needs required for cardiac tissue regeneration, the aim of this research study is to propose lyotropic liquid crystals (LLC) as safe matrices for cardiac application. LLCs have already been investigated for tissue regeneration, showing promising results in the case of several applications [24,25,26]. They are defined as systems whose structural organization is influenced by the amount of solvent [27], and not by other stimuli such as temperature, pH or light induction.

Some polar lipids respond to an excess of water by self-assembling into liquid crystals [28]. Liquid crystals can assume different internal structures called cubic and lamellar phases, as a function of water content. The lamellar phase is characterized by planar lipid bilayers separated by water layers, forming a one-dimensional network. Its low viscosity is a consequence of poor water content. On the contrary, cubic liquid crystalline phases are characterized by an extremely high viscosity due to the water absorption that occurs when the lamellar phase is placed in fluid excess. Cubic phases owe their name to the cubic symmetry visible by X-ray diffraction patterns [27,29], and they show a complex spatial organization. The most important characteristic of the cubic phases in the fully hydrated form is their stability to variations in temperature (from room to body temperature and vice versa), pH [30] and exposure to biological fluids [27].

Both systems, lamellar and cubic phase, are characterized by amphiphilic features, thanks to the presence of both lipid hydrophobic and aqueous hydrophilic domains. Due to this property, cubic and lamellar phases are able to contain, within their structures, drugs characterized by hydrophilic, hydrophobic and amphiphilic nature, making them versatile drug delivery systems [31,32,33].

Since post-myocardial infarction heart failure can be considered a degenerative disease where myocyte loss outweighs any regenerative potential, regenerative biology and tissue engineering can provide effective solutions to repair the infarcted failing heart through the development of drug/stem cell delivery systems. To this aim, here we presented a glycerol monooleate-based lamellar phase applied on cardiac tissue, that responds to contact with physiological fluids, instantly becoming a stable, safe, and degradable cubic phase. Our data demonstrated that LLC is a biocompatible and inert material for myocardial tissue. When applied on the myocardial wall, LLC does not affect the physiological function of the mouse heart and it is biodegraded in a few days. The strength of this work is in the proposed use of an extremely simple and low-cost formulation in terms of quali-quantitative composition, which keeps the possibility open of an easily industrial scale-up. Nile red, fluorescein disodium salt and methylene blue were used as lipophilic, hydrophilic and amphiphilic model drugs, respectively, and the kinetic release profiles of these chemical compounds from in vitro formed cubic phase were evaluated. Moreover, an in vitro degradation test was carried out by using phosphate buffer solution (pH 7.4), evaluating the effects of entrapped drug models on weight loss profiles of LLC. An in-depth rheological study on both the lamellar and cubic phases was carried out to evaluate the influence of temperature, sterilization methods, and stresses on the rheological behavior of systems. From our results, we can affirm that the in vivo obtained cubic phase is perfectly layered on myocardial tissue without altering the normal functionality of the heart and without hindering cardiac dynamic activity. LLC could become a potential novel, relevant biomaterial for myocardial repair and regeneration purposes.

## 2. Materials and Methods

### 2.1. Materials

Glycerol monooleate (GMO, 98 wt% monoglycerides, 1.5 wt% diglycerides, and 0.4 wt% free fatty acids) was provided by BASF Catalysts Germany GmbH (Nienburg, Germany). Absolute ethanol, Tween 80, nile red, fluorescein disodium salt and methylene blue were purchased from Sigma Aldrich s.r.l. (Milan, Italy). All other materials and solvents used in this investigation were of analytical grade. Deionized double-distilled water was used throughout the investigation.

### 2.2. Preparation and Sterilization of LLC Lamellar Phase

The lamellar phase (80:20% *w*/*w* GMO:PBS) was prepared by preheating (45 ± 1 °C) the GMO, and subsequently, the right amount of 10 mM PBS, was added drop by drop. During the addition of aqueous media to GMO, a slight stirring was maintained by using a Magnetic Stirrer RCT Basic (IKA^®^-Werke GmbH & Co. KG Janke & Kunkel-Str. 10 79,219 Staufen Germany/Deutschland).

Lamellar phases containing drug models (nile red, fluorescein disodium salt and methylene blue; 0.5 mg/g) were prepared by solubilizing nile red in pre-heated GMO and by dissolving fluorescein disodium salt and methylene blue in aqueous media, respectively.

To obtain sterilized samples, LLC lamellar phases were sterilized by using an autoclave (International PBI, Milan, Italy) and carrying out cycles of 15 min at 120 °C.

Finally, the in vitro simulated cubic phases were prepared simply by adding an excess of aqueous medium (PBS pH 7.4) to the pre-formulated lamellar phases.

### 2.3. Morphological Analysis of Lamellar and Cubic Phase

Microscopies of lamellar and cubic phases were obtained using the Morphologi G3-S microscope (Malvern Panalytical, UK) equipped with the optical system Nikon^®^ CFI 60 Brightfield/Darkfield. Lamellar phase (10 µL) and cubic phase (10 mg) were loaded one by one on the plate and a micro cover glass (20 × 20 mm, Syntesys, Italy) was applied above the sample to retain the hydration rate. Before starting the analysis, polarized light was obtained by placing crossed polarizing filters (ScreenTech, Germany) above the light source and orienting them to form a 90° angle. Microscope images were acquired using both a 10× or 50× objective, processed using the Morphologi software (Malvern Panalytical, UK) and finally exported as uncompressed TIFF images.

### 2.4. In Vitro Water Uptake

The water uptake of the lamellar phase was investigated using a gravimetric method as shown in another research work [34], with some changes. In detail, 1 mL of LLC lamellar phase was put in a small beaker and was weighed. Subsequently, 5 mL aqueous media (PBS pH 7.4) was added to the lamellar phase. The systems were maintained at 37 ± 0.5 °C to reach the maximum water uptake and swelling equilibrium. After 30 min, the not absorbed excess PBS was removed and the beaker with the obtained cubic phase was weighed. The water uptake (W%) was calculated as reported below [35]:

$$W\%=\frac{Mcp-Mlp}{Mcp}\times 100$$

where *Mcp* was the weight of the beaker with the obtained cubic phase after removing excess water and *Mlp* was the weight of the beaker with pre-formulated lamellar phase.

### 2.5. Rheological Behavior Studies

The rheological behavior of LLC lamellar and cubic phases was evaluated with a Kinexus^®^ Pro rotational rheometer (Malvern Panalytical Ltd., Spectris plc, Malvern (Worcester), UK) using cone-plate geometry (diameter 40 mm; angle 2°; gap between geometries of 1 mm) for lamellar phases and plate-plate geometry (diameter 20 mm; gap between geometries of 1.2 mm) for cubic phases. The temperature was maintained at prefixed values (25 and 37 ± 1 °C) by the Pelter unit.

Each sample was gently put on lower geometry and maintained at rest for 5 min before starting the analysis, to exclude any effect of sample loading [36] on the rheological behavior.

Frequency sweep measurements were carried out on lamellar and on fully swollen cubic phases to record mechanical parameters (G′, G” and ɳ*) at 25 ± 1 °C and 37 ± 1 °C, within a frequency range of 0.1–10 Hz and at a constant shear stress (1 Pa).

Continuous changes (each of 100 s) in strain (100% and 0.5%) were applied on cubic phases to test the strain-induced destruction and recovery of the sample, at 37 ± 1 °C [34].

### 2.6. In Vitro Studies of Model Drugs Release

Drug model-loaded lamellar phases were prepared as previously described by using nile red, fluorescein disodium salt and methylene blue, chosen as lipophilic, hydrophilic and amphiphilic drug models, respectively.

The release profiles were evaluated using the dialysis method. A volume of 1 mL of each LLC lamellar phase formulation was placed in a cellulose acetate dialysis bag (molecular cut-off of 50 kDa) and closed with suitable clips. Each bag was immersed into a beaker containing 100 mL of release medium, which was maintained in continuous stirring and at 37 ± 1 °C. The release media were chosen on the basis of the drug models’ hydrophilicity/lipophilicity; we used PBS pH 7.4 for fluorescein disodium salt and methylene blue and a hydroalcoholic mixture (80:20 H_2_O:EtOH) with 1% Tween80 for nile red. The lamellar-cubic transition occurred within the dialysis bag. At prefixed time intervals after lamellar-cubic transition, 1 mL of release medium was withdrawn and replaced with the same volume of fresh medium. The aliquots were analyzed by using a PerkinElmer Lambda 35 ultraviolet-visible (UV-vis) spectrophotometer at a λmax of 550 nm [37,38], 485 nm [39], and 660 nm [40] for nile red, fluorescein disodium salt and methylene Blue, respectively.

### 2.7. Evaluation of In Vitro Spontaneous Degradation

The in vitro degradation trend was evaluated for each obtained LLC cubic phase (with or without drug models), according to the gravimetric method [41]. In detail, 1 g of lamellar phase was put into a pre-weighed beaker and an excess of degradation medium was added to induce cubic phase formation. When the cubic phase was obtained, the degradation medium (PBS pH 7.4) was replaced with 5 mL of fresh medium previously equilibrated at 37 ± 1 °C. At specific and pre-fixed times, the degradation medium was removed and the beaker containing the cubic phase was weighed and fresh medium was again added to the cubic phase. The weight loss was used as a parameter to indicate the spontaneous degradation grade. The amount of degraded cubic phase was expressed as a percentage and according to the following equation:

$$Weight\;loss\;\left(\%\right)=100-\left[\left(\frac{Wtx}{Wt0}\right)\times 100\right]$$

where *Wtx* was the weight of cubic phase at a fixed time, while *Wt0* was the weight of cubic phase before any addition of degradation media.

### 2.8. Animals

All animal experimental procedures were approved by Magna Graecia Institutional Review Boards on Animal Use and Welfare and performed according to the Guide for the Care and Use of Laboratory Animals from directive 2010/63/EU of the European Parliament. All animals received humane care and all efforts were made to minimize animal suffering.

To evaluate the effects of LCC on myocardial tissue and cardiac function, two-month-old C57BL/6J male mice were used (Jackson Labs, stock number 000664).

Mice were housed under controlled conditions of 25  °C, 50% relative humidity, and a 12 h light (6:00–18:00 h) and 12 h dark cycle, with water and food available ad libitum. Mice were anaesthetised by intraperitoneal (i.p.) injection of ketamine (100 mg/kg) and xylazine (5 mg/kg) or inhaled isoflurane (isoflurane 1.5% oxygen 98.5%, Iso-Vet, Healthcare).

### 2.9. In Vivo Study Design

#### 2.9.1. Echocardiography

Mice were anaesthetized with isoflurane prior to echocardiography analysis and anesthesia was maintained with 1–2% isoflurane in oxygen. Gas was delivered through a nose cone. Unconscious mice were kept under a controlled temperature on a restraining board, weighed and secured in a supine position. Four-limb lead electrocardiograms were simultaneously recorded. Using a depilatory agent, the hair in the thoracic region was removed and the area was cleaned with water. Ultrasound gel was applied to the thoracic region to improve sound wave transmission. The heart rate of the mice was maintained at >400 b.p.m. while images were recorded. Echocardiographic images were obtained using a Vevo 3100 system (Visualsonics, Inc., Toronto, ON, Canada) equipped with an MX550D ultra-high frequency linear array transducer (22–55 MHz) as previously reported [42]. The transducer was positioned perpendicular to the mouse using a stationary stand, alternatively manual adaptations were needed for optimal imaging. When optimal views of the aorta, papillary muscle, and endocardium were visible, the B-mode long-axis parasternal images were recorded. M-mode short-axis images were recorded at the level of the papillary muscles. The left ventricle was bisected to obtain the optimal M-mode selection. Conventional echocardiographic measurements of the LV included ejection fraction (EF), fractional shortening (FS), end-diastolic dimension, end-systolic dimension, anterior and posterior wall thickness, and mass were obtained. EF and FS were calculated by using standard software. Advanced cardiac analysis (regional and global cardiac measurements) was assessed by speckle-tracking echocardiography (Vevo LAB analysis software; VisualSonics). Cardiac cycles were acquired digitally from the parasternal long-axis and mid-ventricular short-axis views for the assessment of radial, circumferential, and longitudinal systolic strain/velocity, and time-to-peak systolic strain/velocity. Images selected for strain analysis had well-defined endocardium and epicardium borders and the analysis was performed according to the manufacturer’s instructions. The endocardium and epicardium were semi-automatically traced using the software VevoStrain. To ensure adequate tracking of endocardium and epicardium borders, the traces were manually adjusted. Velocity, displacement, strain and strain rate were calculated for radial and longitudinal planes. The basal anterior-septum, mid-anterior-septum, apical anterior-septum, basal posterior wall, mid-posterior wall and apical posterior segments in the long axis, were defined. The anterior, anterior-septum, inferior-septum, inferior, posterior and anterior-lateral segments in the mid-ventricular short-axis were further delineated. The tissue contraction patterns were expressed as negative strain values for longitudinal and circumferential motion and positive values for radial strain. In each segment, peak systolic strain (%) and time-to-peak systolic strain (ms) were analyzed.

#### 2.9.2. LLC Application Procedure

The LLC application procedure was performed on 2-month-old C57BL/6J male mice (body weight of 20–22 g). Animals were anesthetized with Tiletamine/Zolazepam, intubated with a 22G tube and ventilated with a mechanical ventilator (28026 mouse ventilator, Ugo Basile, Italy; tidal volume 0.2 mL, 120 strokes/min) while kept at 37 ± 2 °C body temperature. The fourth intercostal space was opened to expose the heart. Mice were treated with 10 μL of either LLC or just saline, injected with a 27½ G needle on the ventral cardiac plane, corresponding to the left ventricle antero-apical myocardial regions. The pneumothorax was then reduced, the chest sutured, and the animals were allowed to recover. Post-surgical analgesia was achieved by buprenorphine (0.1 mg/kg), and ampicillin 150 mg/kg was given at the end of surgery. The animals were divided into different groups based on the experimental arm. Animals were sacrificed and the hearts excised respectively at 1 day, 3 days, 7 days, 14 days and 28 days after LLC or saline control epicardial release.

The acute mortality rate (defined as death by 24 h after the procedure) was overall ~5%.

#### 2.9.3. Tissue Harvesting, Histology, and Immunohistochemistry

In order to perform immunohistochemistry analysis, the abdominal aorta was cannulated, and the heart of each animal was arrested using a solution of cadmium chloride/potassium in diastole. Hearts were perfused through the cannulated aorta and then fixed with 4% PFA or with 10% buffered formalin. The hearts were then cut into apical, mid, and basal regions, and the right and left atria. After being weighed, the LV was sectioned and embedded, respectively, in an Optimal Cutting Temperature Compound or in paraffin. Tissues were cut in 10 µm or 5 µm cross-sections, respectively, and sections were stained with hematoxylin and eosin (H&E) following standard procedures. Cellular apoptosis was detected with the Terminal deoxynucleotidyltransferase (TdT) assay (Roche Applied Science). Dead cardiomyocytes were detected and quantified using fluorescent and confocal microscopy. In order to evaluate CM hypertrophy, a CM cross-sectional area was measured through immunostaining with wheat germ agglutinin (WGA) Alexa Fluor 647 conjugate (1:200 dilution; Invitrogen) and digital analysis of acquired cardiac cross-section images (Leica, 1128 LAS AF Software). CM diameter was measured across the nucleus on three transverse sections (~500 myocytes/animal were sampled). The following primary antibodies were used: monoclonal antibody against cardiac myosin (MF-20, ID: AB_2147781, DSHB), anti-Cardiac Troponin I (1:50 dilution; Abcam). The primary antibody was revealed by respective anti-mouse IgG or anti-rabbit IgG secondary antibody (1:100 dilution; Jackson Immunoresearch). The nuclei were counterstained with the DNA binding dye, DAPI (4,6-diamidino-2-phenylindole, Sigma) at 1 µg/mL.

All stainings were acquired and analyzed using light microscopy (EVOS XL inverted microscopy).

#### 2.9.4. Myocyte Necrosis Analysis

To assess CM necrosis, 2-month-old C57BL/6J male mice were used. Mice received i.p. injection of 100 µg/100 µL of a monoclonal antibody against cardiac myosin (MF-20, ID: AB_2147781, DSHB) 6 h after LCC/saline administration. All animals were sacrificed 1 day after cardiac myosin administration and the heart was fixed with 4% paraformaldehyde (PFA). The number of necrotic MF-20 positive CMs was manually counted in cardiac cross-sections for each power field using a 63X objective for a total of 20 fields and the number of MF-20 positive CMs was expressed as a percent fraction of the total CM number per mm2. All stainings were acquired and analyzed using confocal microscopy (LEICA TCS SP5 and SP8).

### 2.10. Statistical Analysis

Statistical analysis of the various experiments was performed by one-way ANOVA, considering a *p*-value of <0.05 (*) and of <0.001 (**) as significant. A posteriori Bonferroni t-test was carried out to check the ANOVA test.

## 3. Results

### 3.1. Rheological Characterization of Empty Lamellar and Cubic Phases

**Figure 1:**
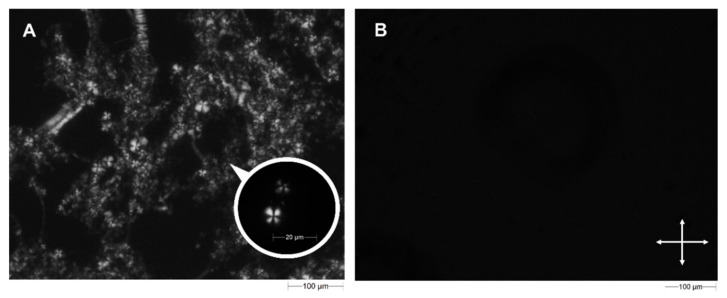
Morphological characterization of lamellar and cubic phases. Microscopies of lamellar (**A**) and cubic (**B**) phases were obtained using the Morphologi G3S equipped with polarizing filters, obtaining the well-known structures referred to both systems. Observations were performed using a magnification of 10× or 50×. White double arrows indicate the orientations of crossed polarizers.

Liquid crystals (LCs) are described as intermediates between crystalline solids and isotropic liquids, namely, in a liquid crystal sample a structural organization and a certain fluidity coexist [43]. The main feature of liquid crystals is their ability to self-assemble in response to specific stimuli; based on stimulus, we can distinguish two types of self-assembling liquid crystals called thermotropic LC, if it changes its state when the temperature is modified, and lyotropic LC, when the sample responds to a change in the solvent amount [44]. In any case, the final result is the in situ gelling process that permits obtaining a solid-like compound near the application site. In situ, gelling systems are an attractive solution for the site-specific delivery of active compounds and for the treatment of several human diseases. The Morphologi G3S is enabled to easily characterize the sample and to screen the final lyotropic liquid crystalline phase. Polarized Light Microscopy (PLM) is frequently used as a crucial tool to determine the optical isotropy of LLC phases. This method allows discriminating the phases which possess the ability of birefringence from those that do not. Quintessentially, the cubic phase is an isotropic phase not visible to polarized light, while the lamellar and hexagonal phases are well known as anisotropic phases which are irrefutably distinguishable according to their typical textures [45]. As can be seen in Figure 1A, microscopies of lamellar phases showed the well known “cruciate flowers”, which are typical for this structure and that allows us to exclude the presence of another mesophase (such as the hexagonal one, identified by a “fan” texture) [46]. On the contrary, no birefringence is observed through the crossed polarizers for cubic phase samples, obtained by adding an excess of an aqueous medium, thus recording a dark background which confirmed the presence of an isotropic stable cubic phase (Figure 1B).

**Figure 2:**
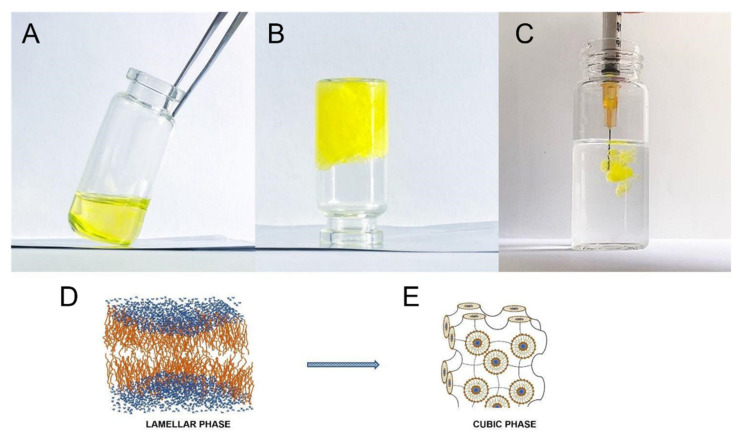
Schematical representation of lamellar and cubic phase. Lamellar (**A**–**D**)–Cubic phase (**B**–**E**) transition of LLCs after exposure of lamellar phase to an excess of PBS (in this case fluorescein disodium salt was added to stain the sample); and representative figure of instantaneous transition (**C**).

In this research work, our attention is focused on in situ forming LLC cubic phase made up of glyceryl monooleate in a simple and low-cost basic formulation; we wanted to propose this formulation as an innovative and biocompatible support to the cardiac tissue when a myocardial infarction occurs. GMO is a polar amphiphilic lipid able to swell in water and form LLCs [47]. The precursor solution, defined lamellar phase, was simply prepared by mixing GMO with a certain amount of aqueous medium (80:20% *w*/*w* GMO:PBS). The in vitro phase transition of lamellar phase to cubic phase was induced, exposing the precursor solution to an excess of hydration medium, namely PBS pH 7.4 (Supplementary Materials Video S1). The occurred phase transition from lamellar to cubic phase was schematically reported in Figure 2.

**Figure 3:**
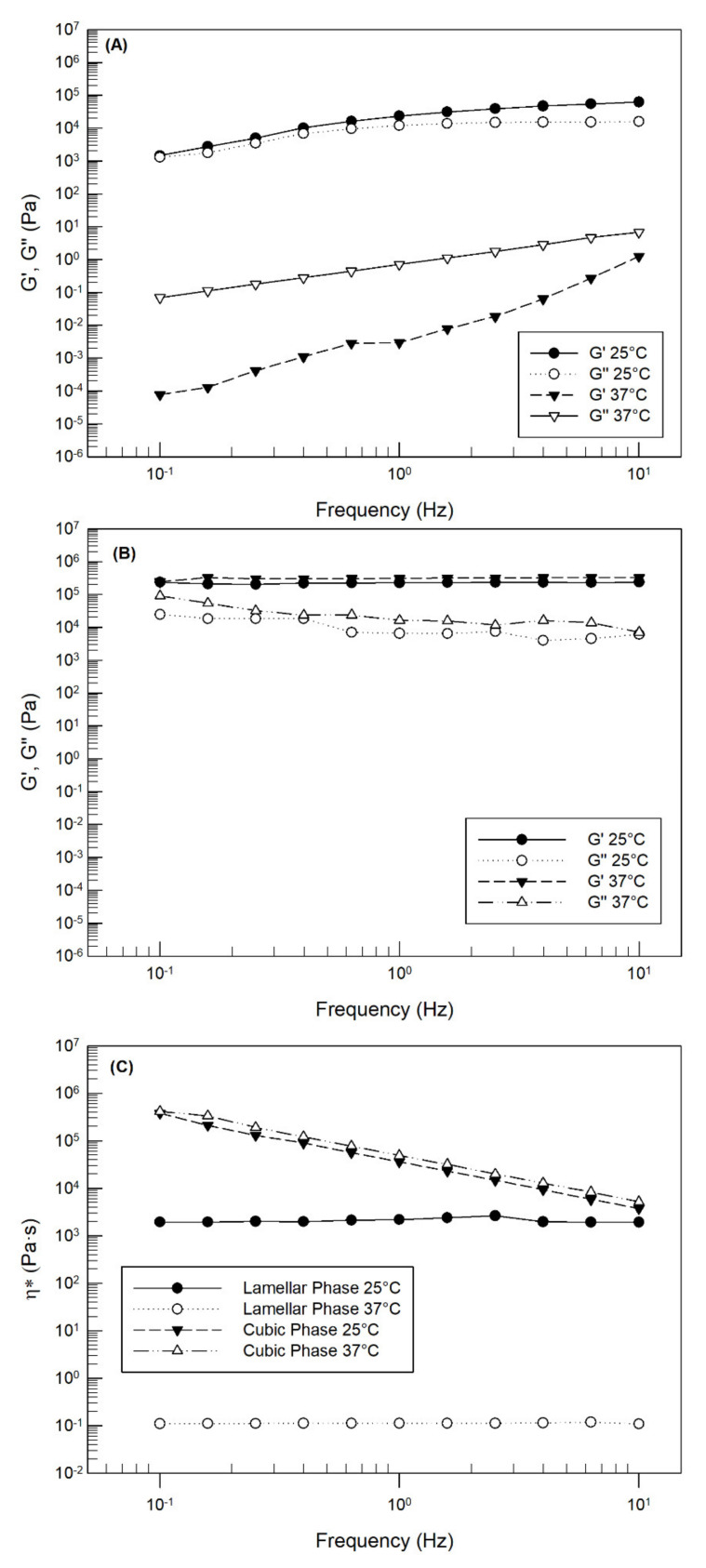
Rheological properties of lamellar and cubic phases were obtained in oscillatory mode by using the Kinexus Rotational Rheometer. Dynamic moduli change of lamellar (**A**) and cubic phase (**B**) and viscosity flow (**C**) of LLC samples were represented as a function of temperature and frequency. The result was a representative experiment of five independent experiments.

The lamellar phase of LLCs was clear and flexible, and it was able to move by tilting the vial (Figure 2A); on the contrary, after exposure to an excess of hydration media, the obtained cubic phase appeared in a gel state, that did not flow as the vial was tilted (Figure 2B). Glyceryl monooleate-based cubic phase is considered thermodynamically stable, optically isotropic, transparent and has a high viscous phase [48]. The cubic phase of LLC consists of a curve bilayer extending in three dimensions and separating two congruent networks of water channels (Figure 2D,E). This inner structure makes the cubic phase akin to both hydrophilic and lipophilic molecules [49]. To distinguish the cubic phase from the lamellar phase, rheological analysis was carried out at 25 °C and 37 °C. Previously, several research groups have demonstrated that the rheological behavior of the lamellar phase was dependent on temperature analysis, while the viscoelastic properties of the cubic phase were not influenced by temperature variation, as shown in Figure 3.

The storage modulus G′ and the loss modulus G″ were evaluated by sweeping the frequency (0.1–10 Hz). The lamellar phase showed a strong temperature dependence, in fact when it was analyzed at room temperature (25 °C), the two moduli were quite overlapping with a slight predominance of G′ (Figure 3A). On the contrary, when the lamellar phase was heated at body temperature (37 °C), its viscoelastic profile was completely changed: G″ > G′ across the entire frequency range tested, indicating a “liquid-like” behavior. This response to heating is an important aspect to define because it allows for an easier application in the lamellar phase on a specific application site, inducing the right stratification and following cubic phase transition. Figure 3B, concerning the viscoelastic profiles of the cubic phase, has confirmed the successful gelation of the sample in the presence of an excess of medium. As shown, the G′ modulus remained almost constant and greater than G″ both with the variation of the frequency and the temperature, indicating the formation of a strong three-dimensional network [50]. Particular attention must be given to Figure 3C, which compares complex viscosity (ɳ*–Pa·s) values of the cubic phase and lamellar phase when they were exposed to different temperatures. First of all, we can note that temperature variation did not alter the rheological behavior of samples, but it induced a marked reduction of lamellar phase viscosity when analyzed at body temperature. If complex viscosity curves of the lamellar phase were influenced by a change of temperature, the viscosity values remained constant over the entire used frequency range, highlighting resistance to the imposed stress, as a Newtonian fluid. This behavior means that the lamellar phase preserved its internal structure during frequency sweep analysis, without flow under applied stress. Contrariwise of lamellar phase and as demonstrated for G′ values, the complex viscosity profile of the cubic phase was not influenced by temperature change: the curves of complex viscosity vs. frequency obtained at 25 °C and 37 °C were overlapped. This trend is a further confirmation of the formation of the lyotropic cubic phase. Analyzing the dependence of complex viscosity from the used frequency range, we can note that the sample responded to a frequency increase with a reduction in ɳ*, adapting its internal structure to the applied solicitation. The variation of cubic phase viscosity as a function of frequency is an important result because it assumes that the cubic phase that will form on the cardiac surface in the presence of biological fluids will be able to adapt to the cardiac movement, without hindering itself and without being unstructured.

**Figure 4:**
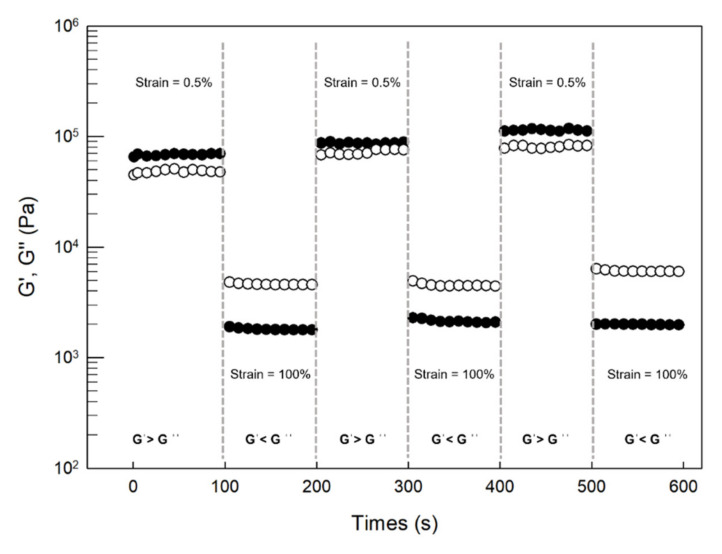
Self-recovery properties of LLC cubic phase analyzed at 37 ± 1 °C. Three cycles of 100% and 0.5% strain (frequency = 1 Hz) were applied on the same sample, evaluating G′ (filled circles) and G″ (empty circles) values. The result was a representative experiment of five independent experiments.

To confirm the ability of the cubic phase of self-recovery, some cycles of large amplitude oscillatory strain (100% of strain at 1 Hz of frequency) and recovery at low strain (0.5% of strain at 1 Hz of frequency) were applied to the cubic phase sample; the results were reported in Figure 4.

At each cycle, the average G′ value of cubic phase markedly decreased when the strain was increased from 0.5% to 100%, meaning that the sample was able to adapt itself at induced deformation (Figure 4). In particular, the increase in strain induced a reverse of G′ and G″ values: when the strain was 0.5%, a superiority of G′ over G″ was recorded at each analysis cycle, highlighting a solid-like behavior of the analyzed sample. In fact, in the presence of low strain, the ordered arrangement of GMO and medium molecules was maintained [34]. Instead, when the strain was increased at 100% (maximum deformation), this ordered inner arrangement of cubic phase was destroyed and the viscoelastic curves of the cubic phase showed an opposite profile with G″ > G′, highlighting a liquid-like behavior.

In any case, when the strain was again brought back to low values (100% → 0.5%), G′ recovered to high values. This step-strain measurement showed a quick recovery of the inner network and confirmed the self-recovery ability of the LLC cubic phase. Rather, since at each analysis cycle the restoration of a strain equal to 0.5% induced a progressive increase in the average values of G′ (Table 1), we can affirm that the continuous stress of the sample induces a continuous strengthening of its structure and that the viscoelastic characteristics were maintained even under disruptive forces. These results are very encouraging considering that the heart is a continuously moving organ; of course, this test cannot simulate the real heart beat but it allows us to hypothesize that thanks to its recovery ability. The LLC can result in a comfortable and conformable strategy for in vivo administration, adapting to eventual deformation of the surrounding tissue [51], as could occur during diastole and systole phenomena [52].

**Figure 5:**
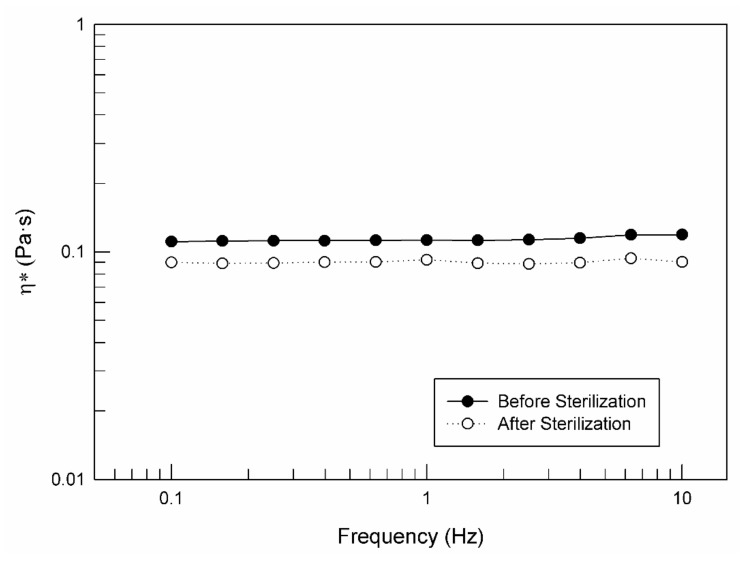
Complex viscosity curves vs. frequency of LLC lamellar phase analyzed at 37 ± 1 °C before and after the sterilization process, carried out by using an autoclave (120 °C for 15 min). The result was a representative experiment of five independent experiments.

Sterilization and its effect on sample properties are important factors to be taken into consideration when a potential in vivo application is supposed. Previous other experimental data reported in the literature confirmed the suitability of this method for sterilizing the monoglyceride-based cubic phase [53]. As shown in Figure 5, the rheological profiles of the lamellar phase were not influenced by the sterilization process carried out in an autoclave (120 °C for 15 min).

In detail, the complex viscosity profile recorded during frequency sweep analysis maintained its trend also after the autoclave process; the only difference that can be seen from Figure 5 is a slight reduction in the complex viscosity values after sterilization. This effect could be advantageous for an easier in vivo application of the lamellar phase.

Moreover, the lamellar to the cubic phase transition of LLC was not influenced by the sterilizing process, in fact, the obtained cubic phases were characterized by the same rheological profiles (data not shown).

### 3.2. Evaluation of Model Drugs Inclusion Effects on Properties of Lamellar and Cubic Phase

**Figure 6:**
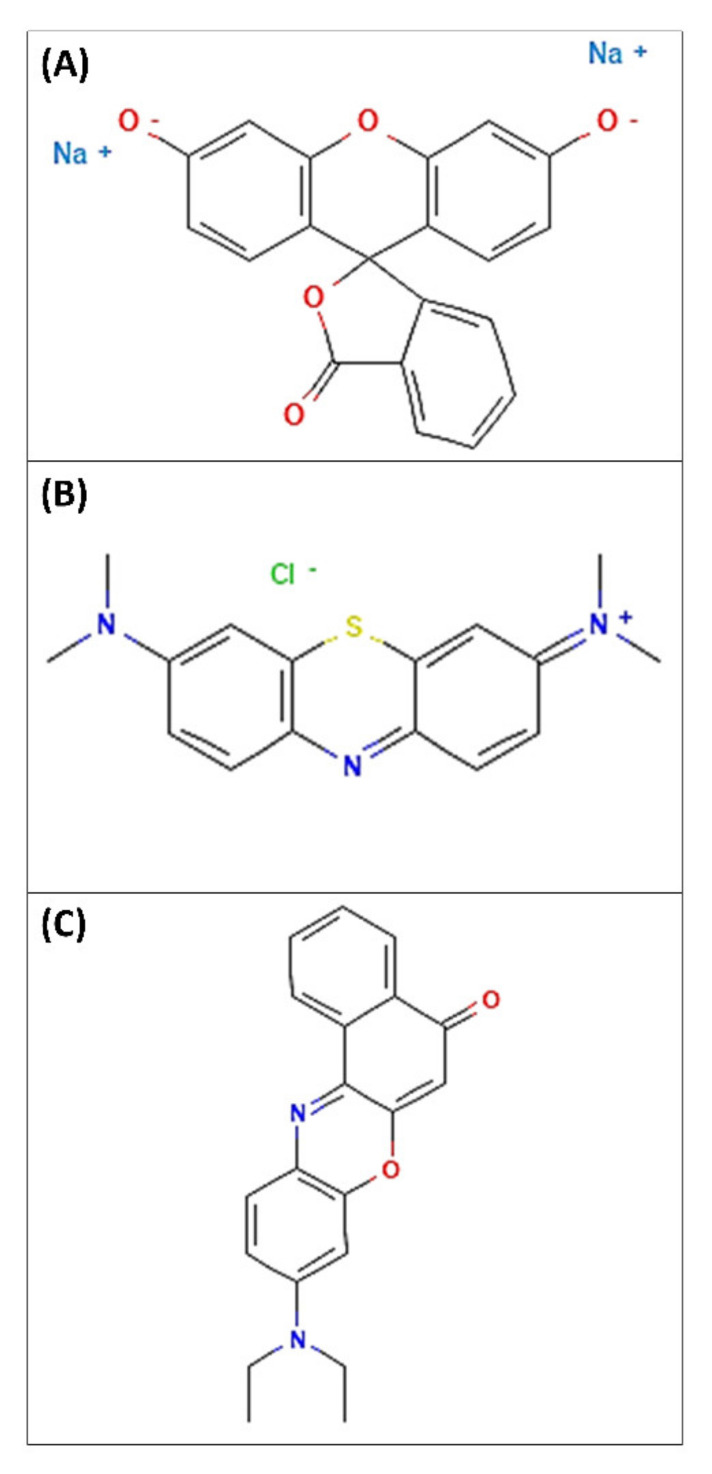
Chemical structures of fluorescein disodium salt (**A**), methylene blue (**B**) and nile red (**C**). https://pubchem.ncbi.nlm.nih.gov/ (accessed on 20 January 2022).

Glyceryl monooleate is able to induce the formation of a physically stable and highly viscous cubic phase starting from a precursor solution, i.e., lamellar phase, in contact with excess aqueous medium [30]. Despite this, the addition of one or more components to these biphasic systems could induce a variation of their specific characteristics [30,54]. Starting from scientific evidence, and supposing a potential application of LLCs as drug delivery systems in post-myocardial infarction treatment, we wanted to analyze the rheological behavior of our samples in the presence of three model drugs, fluorescein disodium salt, methylene blue and Nile ed, characterized by different physicochemical features and chosen as hydrophilic, amphiphilic and lipophilic model drugs, respectively (Table 2, Figure 6).

**Figure 7:**
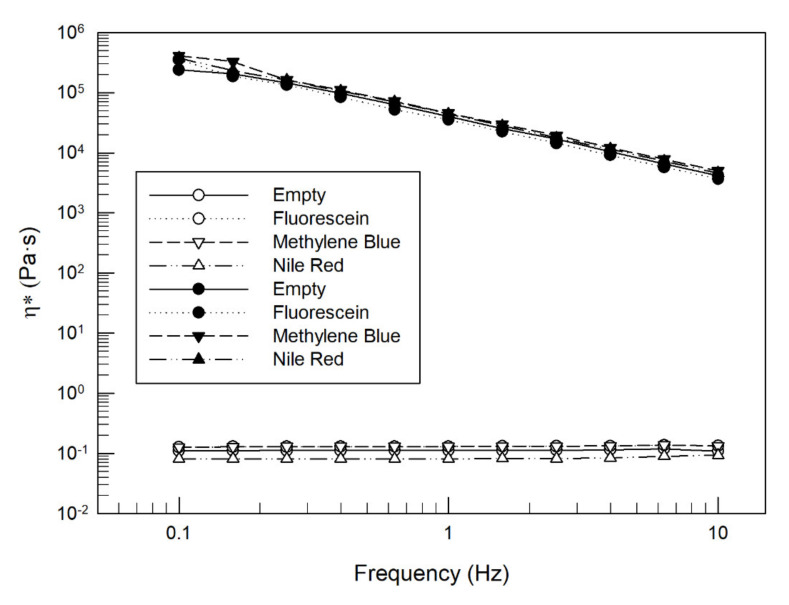
Complex viscosity (ɳ*—Pa·s) curves vs. frequency (Hz) analyzed at 37 ± 1 °C by using the Kinexus Rotational Rheometer for LLC lamellar (empty symbols) and cubic (filled symbols) phases prepared with (0.5 mg/g) and without model drugs. The result was a representative experiment of five independent experiments.

The already demonstrated ability of the lamellar phase to solubilize drugs with different characteristics was confirmed [30,34,58]; in fact, during the lamellar and the cubic phases preparation, no sedimentation or phase separation occurred. The addition of the chosen concentration of each model drug did not influence or alter the transition from lamellar to cubic phase, as shown in Figure 7.

The complex viscosity of the LLC lamellar phase (empty symbols in Figure 7) was maintained almost constantly during all ranges of frequency analysis and around 0.1 Pa·s. The log P of three chosen model drugs did not change the rheological profile of the lamellar phase analyzed at 37 °C, in fact, the curves of complex viscosity vs. frequency were overlapped for all four tested samples. The successful lamellar to cubic phase transition in presence of model drugs, which occurred when the lamellar phase was posed in an excess of PBS pH 7.4, was confirmed by rheological analysis. The cubic phases were characterized by greater complex viscosity in comparison with corresponding lamellar phases, and by a reduction of flow properties as a function of an increase in frequency, exactly as obtained when an empty cubic phase was analyzed (Figure 3C). Moreover, in this case, the curve of complex viscosity vs. frequency of empty and model drugs-loaded cubic phase was overlapped, confirming that the different structural disposition of fluorescein disodium salt, methylene blue and nile red did not alter the viscoelastic properties of our sample. These interesting results encourage future studies because it could be hypothesized to deliver through this in situ cubic phase system a wide range of active molecules with pharmacological activity, without losing the specific feature of GMO-based systems.

The absence of effects of model drugs on lamellar to cubic phase transition was also confirmed by water uptake (Wu%) evaluation. Water uptake can be considered as the ability of the lamellar phase to absorb water, or in our studies PBS pH 7.4, in presence of excess and to increase its weight during its transformation in the cubic phase. Moreover, water uptake values give an idea of the percentage of hydration medium necessary to induce the cubic phase formation, and on the rate of gelling. In this study, a known amount of empty or model drug-loaded lamellar phases was put in a pre-weighed beaker and an excess of PBS was added to it. The cubic phase formation was instantaneous and after 30 min the unabsorbed water was removed, and the values of water uptake were recorded.

As reported in Table 3, the absorption of PBS, and the formation of cubic phase, were not modified when the model drugs were contained into lamellar phase; the differences in weight gain between empty and fluorescein-, methylene blue- and nile red-loaded cubic phase were not statistically significant.

The rheological profiles and the Wu (%) values demonstrated that the incorporation of molecules with different log P did not lead to changes in the inner matrix structure, assuming a solid interaction between the GMO:medium mixture and the used model drugs. This affinity between the tested model drugs and the inner structure of the formed cubic phase was confirmed by release studies results. The cubic phase of LLCs can be considered as a sustained drug delivery system that induced a slow and controlled release over time of drugs, in a manner dependent on physicochemical characteristics of chosen molecules. As shown in Figure 7, the amphiphilic nature of the formed cubic phase tends to retain the embedded drugs for a different time as a function of drug solubility.

**Figure 8:**
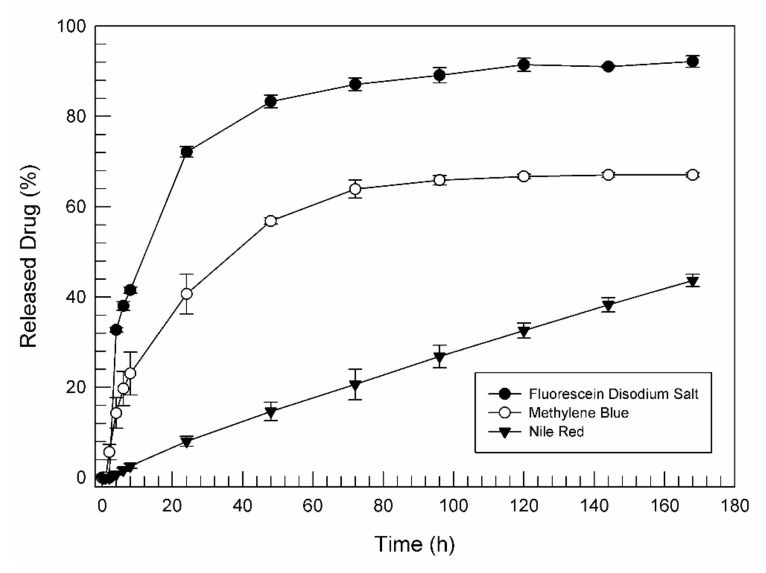
In vitro release of fluorescein disodium salt, methylene blue and nile red from GMO-cubic phase in pH 7.4 buffer solution at 37 °C. The results are expressed as mean values ± standard deviation; the error bar, if not shown, was within the symbol.

The in vitro release of nile red was the lowest one, in comparison with fluorescein disodium salt and methylene blue. The high lipophilicity of nile red probably induced a strong interaction with GMO, becoming an integral part of the lipid component of the network. As shown in Figure 8, a long lag time characterized the lipid drug release, in fact, for the first two hours, no trace of nile red was detected in the acceptor medium. Sink conditions were maintained during the release study and the slow and incomplete release of nile red was not due to poor drug solubility. The release of the drug from the cubic phase was not due to its diffusion through the aqueous channel of the cubic phase, but was due to the progressive degradation of the matrix.

The amount of released fluorescein and nile red at the end of in vitro studies were distinctly different. Thanks to its high hydrophilicity, fluorescein disodium salt was able to easily diffuse through the aqueous channels, obtaining an amount of released drug equal to 72.13 ± 1.18% already after 24 h of experimentation and equal to 92.15 ± 1.25% after 7 days. For nile red, only 8.03 ± 1.09% of the entrapped drug was released during the first 24 h.

From Figure 8, it can be seen that the release profile of the amphiphilic methylene blue has been interposed between the release profiles of the other two dyes chosen. This result confirmed once again the strong influence of the physicochemical characteristics of the drug on the release mechanism from the cubic phases. Methylene blue, characterized by a log P equal to 0.9 [54], probably was able to diffuse through water channels of the cubic phase such as fluorescein but with a lower rate. We can assume that the achievement of release plateau for methylene blue (starting after 72 h of release analysis) is coincidental with a marked degradation of cubic phase (Figure 8).

This data has confirmed the ability of the cubic phase to act as a sustained release drug delivery system, permitting it to obtain a highly controlled and prolonged release of hydrophilic, amphiphilic and lipophilic drugs. In a future clinical application, such a system could reduce the number of drug administrations, improving the patient’s compliance and reducing chirurgical interventions.

**Figure 9:**
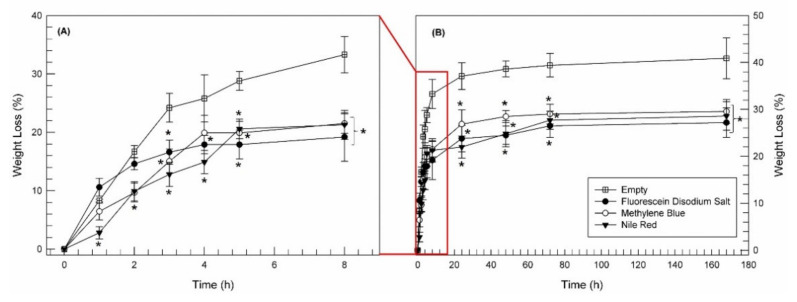
In vitro degradation profiles presented as weight loss (%) curves vs. time (h) for empty and drug-loaded cubic phases. The experiments were carried out in PBS pH 7.4 buffer solution at 37 °C. Panel (**A**) is representative for the first 8 h of entire experiments (**B**). The results are expressed as mean values of five independent experiments ± standard deviation; the error bar, if not shown, was within the symbol. * *p* < 0.05 model drugs-loaded cubic phase vs. empty sample.

The last step of our in vitro characterization of LLC cubic phases concerned their degradation evaluation. While having no effect on the rheological profiles of the cubic and lamellar phases, the addition of molecules with different log P, and their interaction with the structure of lyotropic systems, induced important effects on the in vitro degradation of the cubic phases. The in vitro degradation profiles, shown as weight loss (%) curves vs. time (h), were obtained after exposure of cubic phase to PBS pH 7.4 for a long time, trying to simulate what happens in vivo. As shown in Figure 9, the presence of fluorescein, methylene blue and ile red within cubic structures led to a greater resistance of the cubic phase in the first 8 h of the experiment (panel A), recording a weight loss for the empty cubic phase equal to 33.30 ± 3.11%, in comparison with the weight loss of fluorescein-, methylene blue- and nile red-loaded cubic phase equal to 19.20 ± 4.15%, 21.51 ± 1.65% and 21.30 ± 2.48%, respectively. The difference in degradation behavior between empty and model drugs-loaded cubic phases was maintained during the entire duration of the experiments (1 week, Figure 9B). Despite the different physicochemical features of fluorescein disodium salt, methylene blue and nile red, all chosen model drugs induced a significant reduction (*p* < 0.05) of in vitro degradability of cubic phase, at all tested time points.

### 3.3. In Vivo Studies

#### 3.3.1. LLC Epicardial Application Does Not Affect Myocardial Tissue Viability and Structure

**Figure 10:**
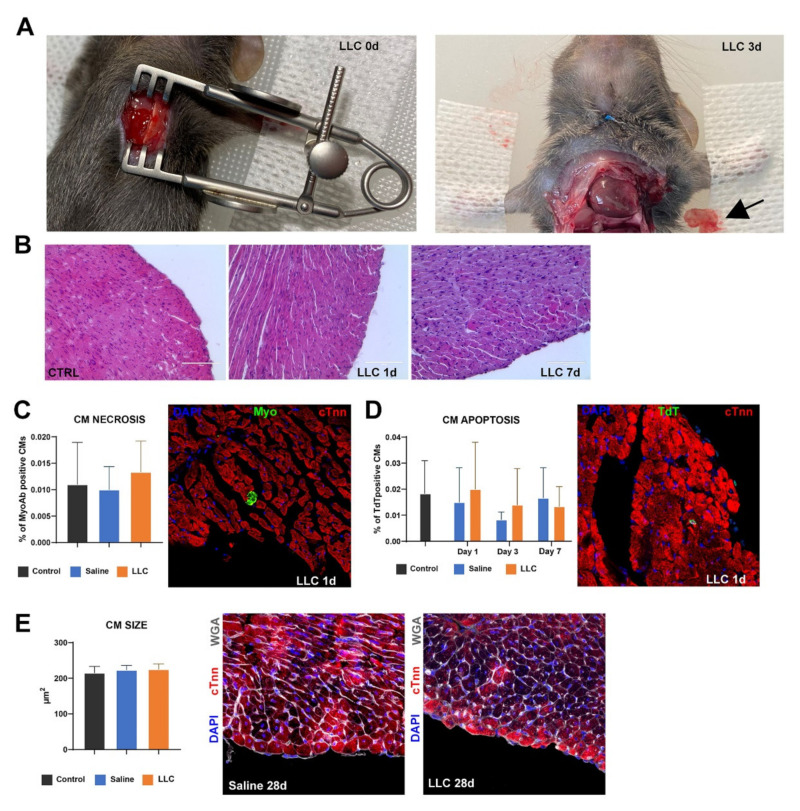
Effects of LLC on myocardial tissue and cardiac function. (**A**) Representative images showing LCC application on the left ventricle antero-apical myocardial regions (left). The LLC was still present 3 days after the application (right; arrow points at the hydrogel removed from the myocardial wall) and was practically completely degraded at 7 days post-application (see Supplementary Materials Video S2 and Video S3). (**B**) Representative H&E stainings of the epicardial region from treated mice 1 day and 7 days after local LLC application as compared with shame-operated control (CTRL) mice. No evident inflammatory reaction or myocardial disarray could be detected. (**C**) Representative confocal image and bar graph of necrotic CMs labelled in vivo with a monoclonal antibody against cardiac myosin (green) 6 h after LLC/saline administration. Mice were sacrificed 24 h after myosin injection. LLC myocardial application did not cause diffuse CM necrosis when compared to the sham-operated control mice and the saline-injected controls. (**D**) Representative confocal image and bar graph of apoptotic CM death at 1, 3 and 7 days after local LCC application. LLC exposure did not cause an increase in apoptotic CM death when compared to saline-injected controls as identified using the TdT (TUNEL) assay with dUTP. (**E**) Representative confocal images and bar graph of a cardiac cross-section showing CM hypertrophy in mice 28 days after LLC application (WGA, wheat germ agglutinin, Cy5 staining, and white fluorescence; cTnn, red; DAPI, blue nuclei). No CM hypertrophy was been detected both in control saline- as well as LLC-injected hearts. All data are mean  ±  SD.

To evaluate the effects of LLC per se on myocardial tissue and cardiac function, thirty 2-month-old C57BL/6J mice were submitted to left thoracotomy followed by pericardiectomy to expose the heart. Control mice were treated with the application of 10 μL of either LLC or just saline, injected with a 27½ G needle on the ventral cardiac plane, corresponding to the left ventricle antero-apical myocardial regions. The LLC was maintained at ∼37 °C for ∼3 min prior to injection for an easy withdrawal, considering that rheological studies (Figure 3C) had proved a reduced viscosity of LLC lamellar phase when it was maintained at 37 °C. Mice were sacrificed and the hearts were excised respectively at 1 day (*n* = 6), 3 days (*n* = 6), 7 days (*n* = 6), 14 days (*n* = 6) and 28 days (*n* = 6) after LLC or saline control epicardial application. Three sex- and age-matched mice were sacrificed at day 0 before the LLC application and were used as baseline sham controls. Despite the results obtained from an in vitro degradation test, gross anatomy showed that the LLC was visible at 1 day, still present at 3 days but practically completely biodegraded already after 7 days post-application (Figure 10A). This result confirmed the biodegradable properties of LLCs, probably assured in vivo by the presence of enzymatic degradable systems and heart movements. No evident inflammatory reaction or myocardial disarray has been detected in the epicardial region from mice after 1 day and 7 days from the local hydrogel release (Figure 10B). To quantify cardiomyocyte (CM) necrosis, 6 h after LLC/saline administration randomized mice from the 1 day group were injected with a monoclonal antibody against cardiac myosin [42] and were sacrificed 24 h (i.e., 1 day).

LLC application did not cause significant diffusion or focal CM necrosis as revealed by in vivo myosin antibody labelling when compared to saline-injected controls (Figure 10C). Myosin-labelled CMs showed clear morphologic features of necrosis with loss of cell membrane integrity and architectural disarray (Figure 10C). Necrotic damage was seldomly detected only in the epicardium of both LLC and saline-treated mice (Figure 10C). Concurrently, LLC exposure did not cause an increase in apoptotic CM death when compared to saline-injected controls as identified using the TdT (TUNEL) assay with dUTP (Figure 10D). The number of apoptotic TdT labelled CMs were indeed minimal, if not trivial, both in the left ventricle of control saline-injected animals as well as of LLC-treated animals at 1, 3 and 7 days after the surgical procedure (Figure 10D). Apoptotic CM death was confirmed by caspase-3 labelling (data not shown). Finally, in line with the data on myocardial cell damage, 28 days after surgical procedure, there was no CM hypertrophy both in control saline- as well as LLC-injected hearts (Figure 10E).

Overall, this data shows that the LLC is a biocompatible and inert material for myocardial tissue, that it is biodegraded in vivo in a controlled time window, making it a potential novel and relevant biomaterial for myocardial repair and regeneration purposes.

#### 3.3.2. LLC Epicardial Application Does Not Affect Global and Regional Left Ventricular Physiological Performance

To assess the functional consequences of LLC epicardial application on left ventricular performance, all animals described above underwent baseline (Base) echocardiography (ECHO) prior to LLC/Saline injection and at 1, 2, 7, 14 and 28 days thereafter. Saline-injected mice were used as sham controls. As saline did not affect any of the ECHO parameters over 28 days and was similar to the baseline of the other groups (data not shown), these animals were not included in the inter-groups analysis.

**Figure 11:**
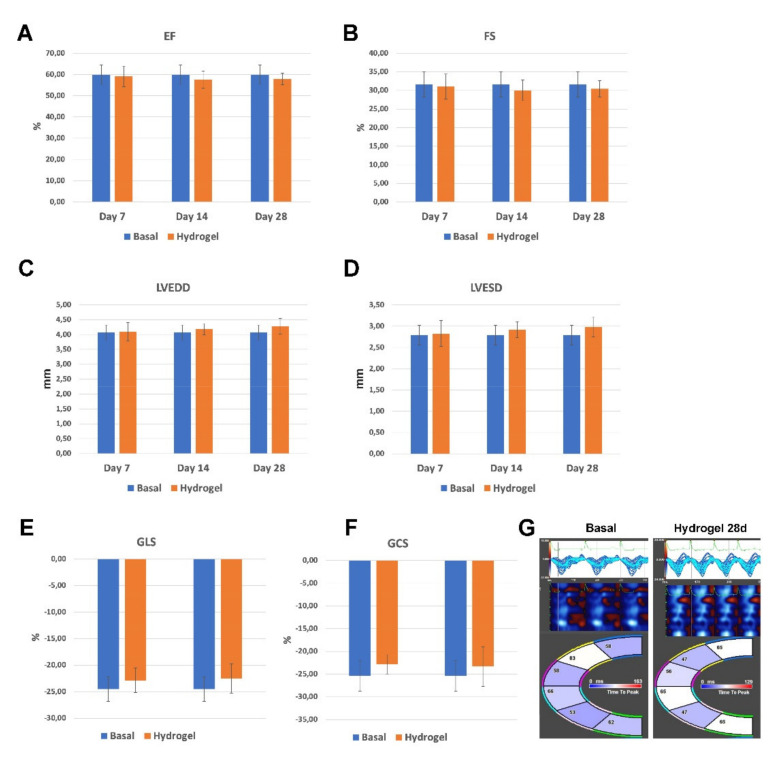
Effects of LLC on global and regional left ventricular physiological performance. (**A**,**B**) Cumulative data of cardiac function at 7, 14 and 28 days after LLC application. LLC application did not change ejection fraction (EF) and fractional shortening (FS). (**C**,**D**) Cardiac dimensions were unchanged in 7, 14 and 28 days LLC treated mice as compared to their baseline as revealed by the measurement of the left ventricular end diastolic diameter (LVEDD) (**C**) and the left ventricular end-systolic diameter (LVESD) (**D**). (**E**,**F**) Cumulative data of global longitudinal and circumferential strain (GLS (**E**) and GCS (**F**), respectively) at baseline and 28 days after LLC application. Global strain analysis did not detect any subtle changes in cardiac muscle performance at 28 days after LLC application when compared to baseline. (**G**) Representative images of regional speckle-tracking strain analysis throughout four segments (apical, anterior and inferior segments wall, and mid-anterior and inferior wall) at baseline and 28 days after LLC application. Regional analysis 28 days after LLC epicardial application showed no difference when compared to baseline. All data are mean  ±  SD.

Considering the liquid phase of LLC, its application on the epicardial wall created an artifactual hypoechogenic cardiac area that significantly impaired the echocardiographic window, making impossible a quantitative evaluation of cardiac function at 1 and 3 days after LLC application (see baseline echocardiographic view in Video S2 as compared to the echocardiographic acquisition of the same animal at 1 day after LLC application in Video S3). Consistently with the above anatomy data, the degradation of the LLC by 7 days allowed the echocardiographic imaging analysis from this time point. As compared to baseline, the LLC application did not change ejection fraction (EF) at 7 days (59.9 ± 4.95% vs. 59.1 ± 4.86%; p = NS) through 28 days (59.9 ± 4.95% vs. 57.88 ± 3.97%; p = NS) (Figure 10A). Concurrently, as compared to baseline, the LLC application did not change fractional shortening at 7 days (31.6 ± 3.36% vs. 31.03 ± 3.85%; p = NS) through 28 days (31.6 ± 3.36% vs. 30.43 ± 2.16%; p = NS) (Figure 11B). The above surrogate measures of cardiac function analysis were reflective of the unchanged cardiac dimensions as revealed by the measurement of left ventricular end diastolic diameter (LVEDD) and left ventricular end systolic diameter (LVESD). Indeed, as compared to baseline, the LLC application did not change LVEDD at 7 days (4.07 ± 0.2% vs. 3.75 ± 0.31%; p = NS) through 28 days (4.07 ± 0.19% vs. 4.28 ± 0.26%; p = NS), as well as it did not change LVESD at 7 days (2.79 ± 0.24% vs. 2.50 ± 0.30%; *p* = NS) through 28 days (2.79 ± 0.24% vs. 2.99 ± 0.24%; *p* = NS) (Figure 11C,D).

Standard echocardiographic measurements are relatively insensitive to subtle changes in cardiac performance, particularly in mice [42,59]. Therefore, to directly measure myocardial contractility and the effects of the LLC on global and regional myocardial function, speckle-tracking-based strain analysis on long-axis and short-axis B-mode was performed [42,59]. Global strain analysis did not detect any subtle changes in cardiac muscle performance at 28 days after LLC application when compared to baseline values (Figure 10E,F). Indeed, the global longitudinal strain was practically unchanged at 28 days when compared to baseline (−24.53 ± 2.30% vs. −22.51 ± 2.73%; p = NS) (Figure 11E). Similarly, the global circumferential strain was not significantly affected by the LLC at 28 days when compared to baseline (−25.34 ± 2.1 vs. −23.32 ± 4.36%; p = NS) (Figure 11F).

Finally, to further evaluate the effects of the LLC on regional myocardial function, regional speckle-tracking strain analysis throughout four segments was performed. Regional analysis at 28 days after LLC epicardial application showed no difference when compared to baseline (Figure 11G).

Overall, this data shows that the LLC application on the myocardial wall does not affect the physiological function of the mouse heart. Together with the histology analysis above, this data provides compelling evidence for the LLC as a potential novel, relevant biomaterial to locally release cells or biomolecules for myocardial repair and regeneration purposes. As for the latter, the presented data did not specifically address the effects of the epicardial application of the LLC on cardiac remodeling in a relevant model of cardiac injury such as myocardial infarction. Nevertheless, the present data indirectly predict that the LLC would be inert at a minimum when applied on the infarcted myocardial wall. Indeed, preliminary data confirm this hypothesis and are currently ongoing.

## 4. Conclusions

The results of this study show the actual applicability of lyotropic liquid crystals for cardiac tissue. We have demonstrated that the rheological characteristics of our scaffold are suitable for supporting the cardiac tissue, and they are not influenced by the presence of any drugs with different physicochemical features. Moreover, the actual liquid—cubic phase transition has been confirmed by the marked change in viscosity values, highlighting the instant increase in cubic phase viscosity. Specific rheological tests were carried out to evaluate the ability of LLC to support and bear heart movement, and the results showed that LLC does not undergo an irreversible de-structuring but, on the contrary, restore their structure at the end of each stress. Despite the high viscosity shown by the cubic phase, in vivo studies have shown that LLC does not induce suffering to the mice and does not induce significant alterations in the anatomy and function of the heart tissue, confirming the biocompatibility and the inertia of LLC for cardiac tissue. All the results obtained from in vitro and in vivo studies encourage the use of LLC as a possible new biomaterial for myocardial repair and regeneration.

## Figures and Tables

**Table 1:** Average G′ values of cubic phase, subjected to repeated cycles of high strain (100%) and low strain (0.5%), at 37 ± 1 °C and at a constant frequency of 1 Hz. * *p* < 0.05 and ** *p* < 0.001 vs. first cycle (0–200 s).

Cycle	Average G′ Value (kPa) (Strain = 0.5%)	Average G′ Value (kPa) (Strain = 100%)
0–200 s	68.30 ±1.54	1.81 ± 0.04
200–400 s	87.26 ± 1.24 **	2.14 ± 0.08 *
400–600 s	114.11 ± 2.36 **	1.99 ± 0.01 *

**Table 2:** Model drugs, their structure and physicochemical properties.

Drug	MW ^a^ (g/mol)	Log P	References
Fluorescein Disodium Salt	376.3	−1.52	[55]
Methylene Blue	319.85	0.9	[56]
Nile Red	318.4	5	[57]
	data	data	

^a^ MW = molecular weight.

**Table 3:** Water Uptake (Wu%) values were obtained for empty and model drugs-loaded lamellar phase during its cubic phase transformation. The results are expressed as mean values of five independent experiments ± standard deviation. The differences between the obtained results are not statistically significant.

Lamellar—Cubic Phase	Wu (%)
Empty	85.55 ± 1.83
Fluorescein Disodium Salt	89.26 ± 2.43
Methylene Blue	86.28 ± 2.60
Nile Red	80.20 ± 2.90

## Data Availability

The data presented in this study are available from the corresponding author upon request. The data are not publicly available due to privacy.

## Supplementary Materials

The following supporting information can be downloaded at: www.mdpi.com/article/10.3390/pharmaceutics14020452/s1, Video S1: instant gelation of lamellar phases; Video S2: echocardiographic window day one; Video S3: echocardiographic window day three.
