# Supplementary material for: Lyotropic Liquid Crystals: A Biocompatible and Safe Material for Local Cardiac Application
Source: Pharmaceutics. 2022 Feb 20;14(2):452. doi: 10.3390/pharmaceutics14020452 (PMC8879243; doi:10.3390/pharmaceutics14020452)
Supplement: Supplementary file 1 [file pharmaceutics-14-00452-s001.zip › pharmaceutics-1585332/pharmaceutics-1585332-sup.docx]

Supplementary Materials: Lyotropic Liquid Crystals: A Biocompatible and Safe Material for Local Cardiac Application

Antonia Mancuso, Eleonora Cianflone, Maria Chiara Cristiano, Nadia Salerno, Martine Tarsitano,
Fabiola Marino, Claudia Molinaro, Massimo Fresta, Daniele Torella and Donatella Paolino

Videos’ Leggend:

**Video S1:** Representative transition of lamellar phase in cubic phase.

**Video S2:** Representative echocardiographic view in long axis of a mouse heart at baseline before LLC application.

**Video S3:** Representative echocardiographic view in long axis of the mouse heart at 1 day after LLC application. It is evident the hypo/ana- echogenic area of the LV created by the application of the LLC, which makes it impossible to perform standard two dimensional and mono-dimensional echo analysis.
